# A novel algorithm for finding optimal driver nodes to target control complex networks and its applications for drug targets identification

**DOI:** 10.1186/s12864-017-4332-z

**Published:** 2018-01-19

**Authors:** Wei-Feng Guo, Shao-Wu Zhang, Qian-Qian Shi, Cheng-Ming Zhang, Tao Zeng, Luonan Chen

**Affiliations:** 1 Key Laboratory of Information Fusion Technology of Ministry of Education, School of Automation, Northwestern Polytechnical University, Xi’an, 710072 China; 2 Key Laboratory of Systems Biology, Institute of Biochemistry and Cell Biology, University of Chinese Academy of sciences, Shanghai, 200000 China; 3 School of Life Science and Technology, ShanghaiTech University, Shanghai, 200000 China

**Keywords:** Target control, Objectives-guided optimization, Drug targets, Dynamics of complex networks

## Abstract

**Background:**

The advances in target control of complex networks not only can offer new insights into the general control dynamics of complex systems, but also be useful for the practical application in systems biology, such as discovering new therapeutic targets for disease intervention. In many cases, e.g. drug target identification in biological networks, we usually require a target control on a subset of nodes (i.e., disease-associated genes) with minimum cost, and we further expect that more driver nodes consistent with a certain well-selected network nodes (i.e., prior-known drug-target genes).

**Results:**

Therefore, motivated by this fact, we pose and address a new and practical problem called as target control problem with objectives-guided optimization (TCO): how could we control the interested variables (or targets) of a system with the optional driver nodes by minimizing the total quantity of drivers and meantime maximizing the quantity of constrained nodes among those drivers. Here, we design an efficient algorithm (TCOA) to find the optional driver nodes for controlling targets in complex networks. We apply our TCOA to several real-world networks, and the results support that our TCOA can identify more precise driver nodes than the existing control-fucus approaches. Furthermore, we have applied TCOA to two bimolecular expert-curate networks. Source code for our TCOA is freely available from http://sysbio.sibcb.ac.cn/cb/chenlab/software.htm or https://github.com/WilfongGuo/guoweifeng.

**Conclusions:**

In the previous theoretical research for the full control, there exists an observation and conclusion that the driver nodes tend to be low-degree nodes. However, for target control the biological networks, we find interestingly that the driver nodes tend to be high-degree nodes, which is more consistent with the biological experimental observations. Furthermore, our results supply the novel insights into how we can efficiently target control a complex system, and especially many evidences on the practical strategic utility of TCOA to incorporate prior drug information into potential drug-target forecasts. Thus applicably, our method paves a novel and efficient way to identify the drug targets for leading the phenotype transitions of underlying biological networks.

**Electronic supplementary material:**

The online version of this article (doi: 10.1186/s12864-017-4332-z) contains supplementary material, which is available to authorized users.

## Backgroud

One final goal of our efforts is to control the complex systems in our daily life. In the past decades, plenty of attentions [1–5] have been paid into the study of the structures and dynamics of complex networked systems, especially biological systems. A frontier area of the research in network science and engineering is controlling complex networks, such as biological molecule networks. Since the law of some systems is hidden, it is difficult to study the controllability of the nonlinear systems directly, especially for the large scale biological systems. However, it is possible to obtain local analytical results for the controllability of nonlinear systems by developing control schemes of linear dynamic systems. Nearly decades of efforts on the controllability of linear dynamic networks, not only review a sufficient condition for “local controllability” of a nonlinear system about a trim point but also result in tremendous advances in our understanding of the problem of controlling complex networked dynamical systems [5–9]. In a recent breakthrough, an efficient algorithm with low polynomial time was provided for computing the minimal quantity of input nodes needed to control any given large-scale directed network [6]. But, it was also shown that in the case of sparse inhomogeneous networks, such as most of the networks emerging from biochemical and biomedical applications, controlling the entire system is expensive. On the other hand, in terms of practical applications in many cases, it is enough to control only a certain well-selected portion of the network’s nodes, such as the set of essential genes, in order to impose a certain overall behaviour over the system. Thus, an interesting question, known as target control problem of complex networks, is posed that how can we chose the driver variables from the system to control a subset of the whole nodes (or a subsystem) about a trim point [8].

However, the traditional framework of network control can only be applicable for the simple networks, and it can not address the target control problem of the large scale of networks. To solve the problem, Wu et al. has proposed a method to solve the target control problem by constructing a weighted bipartite network [10]. But this method may fail when there does not exist a perfect matching in most cases. Meanwhile, Gao et al. proposed another method which offers an approximation on the minimum set of input nodes for target controlling the networks [8]. However, the above researches only focus on controlling the system through any minimum driver-node set and ignore the existence of multiple candidate driver-node sets for control a targeted subset of the network. When we actually expect to control the system with objectives optimization, the different driver-node sets may not participate in target control equally. This consideration prompts us to study how to find the desired solution for target controlling complex networks with objectives optimization. A practice of this consideration can come from our aim for combinatorial drug target identification: we not only consider how to control the disease-associated genes with the minimum driver nodes, but also expect that more driver nodes can be consistent with the set of well-known drug-target genes. Here, we pose a new target control problem with the objectives-guided for finding the optimal driver nodes that minimize the total quantity of drivers and also maximize the quantity of constrained nodes within the drivers.

In this paper, we develop a novel algorithm (TCOA) to identify the drivers for efficiently controlling targets in complex networks. Our algorithm consists of three steps: We first construct the target control tree of the network by finding the maximum matching in the constructed iterated bipartite graph or “linking and dynamic graph” and identify the controllable targets of each node by obtaining its reachable target nodes in the control tree; Then we find the set of optional driver nodes by using the integer linear programming to optimize a regulated factor, which is introduced to balance the quantity of driver nodes and the quantity of driver nodes within the set of constrained (or pre-selected) nodes; Finally we define the maximum matching of the constructed iterated bipartite graph or “linking and dynamic graph” as a Markov chain and use a Markov chain Monte Carlo (MCMC) approach to sample from the sets of all possible maximum matching. We have evaluated TCOA on several real-world networks, and the experiment results support that TCOA outperform existing control-focus approaches. Especially, we have also applied TCOA algorithm to analyze the PPI signaling transduction networks in pancreatic cancer, and Inflammatory bowel disease network from KEGG. The results further illustrate that our TCOA can efficiently identify the driver nodes with more optional property to guarantee the system target controllable, compared to several control-focus approaches. In addition, the experiment results on the two biological cases can also supply an efficient bioinformatics tool to identify the drug targets for leading the phenotype transitions of underlying biological networks.

## Methods

### Problem formulation

Since the law of the some network dynamics, such as the biological networks is hidden, it is difficult to directly study the controllability of the nonlinear networks. Most complex systems are characterized by nonlinear interactions between the components, and usually local properties can be verified [11, 12]. Thus, it is possible to obtain local analytical results for the controllability of nonlinear systems [13, 14]. Here, we review a sufficient condition for “local controllability” of a nonlinear system about a trim point. A system is “locally controllable” if there exists a neighborhood in the state space such that all initial conditions in that neighborhood are controllable to all other elements in the neighborhood with locally bounded trajectories [15]. Considering a dynamic system governed by a set of ordinary differential equations,

1 $$ \left\{\begin{array}{l} dx/ dt=f(x)+\mathbf{B}u\\ {}y=\mathbf{C}x\end{array}\right. $$

where the function *f*(*x*) denotes the system’s dynamics. *x* ∈ *R*^*N*^ and *y* ∈ *R*^*NO*^ represent nodes state and target (output) nodes state; The element ***B***_*ij*_ in **B**∈ *R*^N*NC^ represents whether the node *v*_*i*_ among *V* = {*v*_1_,*v*_2_,…,*v*_*N*_} is inputed by the *j*-th signal [16–18]*.* **C**∈*R*^NO*N^ represents the output matrix.

We are interested in how to find proper matrix ***B*** to gurantee the system (1) locally target (or output) controllable through the input *u* = [*u*(1),*u*(2),..,*u*(*NC*)]. Let *x*_0_ be defined as, *f*(*x*_*0*_) = 0,*A*(*x*_*0*_)=*∂f*(*x*_*0*_)/*∂x* and *G*(*x*_*0*_) *=* [*CB CAB* …*CA*^*N*-*1*^*B*], where *f*(*x*_*0*_) = 0 provides the system’s steady state, *A*(*x*_*0*_) represents the system’s local (linear) dynamics around a trim point *x*_0_ and *G*(*x*_*0*_) guarantees the system local target controllable. The dynamics in (1) are locally target controllable around *x*_*0*_ if rank (*G*(*x*_*0*_)) = *NO* [13, 14]. Therefore the local target controllability analysis of (1) about a trim point therefore reduces to the linear target controllability analysis of (2),

2 $$ \left\{\begin{array}{l} dx/ dt=A\left(x-{x}_0\right)+\mathbf{B}u\\ {}y=\mathbf{C}x\end{array}\right. $$

The dynamics in Eq. 1 are deemed “locally structurally target controllable” if the linearized dynamics in Eq. 2 are structurally target controllable. And the linearized dynamics in Eq. 2 is structurally target controllable if the follow equation is satisfied when we can choose the non-zero values in *A* and *B*,

3 $$ \max \left\{\mathit{\operatorname{rank}}\left[\mathbf{CB},\kern0.5em \mathbf{CAB},\kern0.5em {\mathbf{CA}}^{\mathbf{2}}\mathbf{B},\dots, \kern0.5em {\mathbf{CA}}^{N-1}\mathbf{B}\right]\right\}= NO $$

In a given directed network with nodes *V* = {*v*_1_,*v*_2_,…,*v*_*N*_}, let *O* and *D* be the set of target nodes and the driver nodes, assuming that we expect more driver nodes could be constrained in a set *Q*, where both *O* and *D* and *Q* are the subset of *V*. The output matrix and the input matrix can be set as ***C*** = [***I***(1); ***I***(2);…***I***(*NO*)] and ***B*** = [***I***(*b*_1_); ***I***(*b*_2_);…***I***(*b*_*d*_)]; ***I***(*i*) represents *i*-th row of N*N unit matrix ***I,*** {*b*_1_,*b*_2_,…,*b*_*k*_} is the index of identified driver nodes set *D*. For the purposes of this work, the adjacency matrix of the network is used to find the structure of *A* in Eq. 2. Given the constrained nodes set *Q*, we focus on how to find a suitable driver nodes set *D* such that

4 $$ {\displaystyle \begin{array}{l}\min {f}_1=\left\Vert D\right\Vert \kern0.5em \\ {}\max {f}_2=\left\Vert Q\cap D\right\Vert \kern0.5em \\ {}s.t.\max \left\{\operatorname{rank}\left(\left[\mathrm{CB},\mathrm{C}\mathrm{AB},\mathrm{C}{\mathrm{A}}^2\mathrm{B},\dots, \mathrm{C}{\mathrm{A}}^{N-1}\mathrm{B}\right]\right)\right\}= NO\end{array}} $$

where ||*D*|| denotes the quantity of nodes in the identified driver nodes set *D* and ‖*Q* ∩ *D*‖ represents the quantity of drivers in the constrained or pre-selected nodes set *Q*; the objective functions *f*_1_ and *f*_2_ aim to find the optional drivers with minimum quantity of drivers *D* and maximum quantity of drivers in the pre-selected nodes set *Q* respectively.

**Fig. 1:**
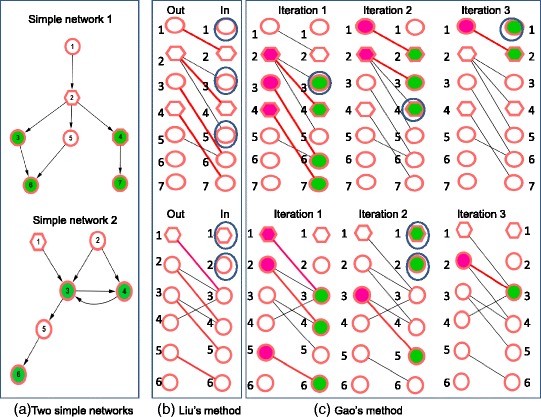
Demonstration of the limitations of the existed methods for the target control problem with objectives-guided optimization. **a** Two simple networks. In the two networks, the target set is {*v*_3_,*v*_4_, *v*_6_,*v*_7_} and {*v*_3_,*v*_4_, *v*_6_} respectively (highlighted in green) and the constrained nodes set is {*v*_2_,*v*_4_} and {*v*_1_} respectively (shape in hexagon). Here we want to minimum the quantity of driver nodes to control the target nodes set {*v*_3_,*v*_4_, *v*_6_,*v*_7_} and {*v*_3_,*v*_4_, *v*_6_} (i.e., disease-associated genes) and maximum the identified driver nodes within the constrained nodes (i.e., practical constraints as prior known drug targets). **b** By applying full control of Liu’s method to the two networks, we can identify the unmatched nodes {*v*_1_,*v*_3_,*v*_5_} and {*v*_1_,*v*_2_} (nodes within the blue circle) in the right side of the bipartite graph transferred from the directed network, as the driver nodes(more details seen in ref. [6]). **c** By using target control of Gao’s method, they first obtain the updated bipartite graph by choosing the nodes in the left side in the previous matching (highlighted in grape) as the nodes in the right side of the current matching (highlighted in green) and then calculate a maximum matching in the updated bipartite graph. Finally they add unmatched nodes (nodes within the blue circle) in right side of the updated bipartite graph to the set of driver nodes (more details seen in ref. [8]), which identify the set of driver nodes of the two networks as {*v*_1_,*v*_3_,*v*_4_} and {*v*_1_,*v*_2_}. In the simple example 1, according to the k-walk theory in Re. [8], it is easy to know that node *v*_2_ can control *v*_3_ and *v*_6_ and *v*_4_ can control *v*_4_ and v_6_. For the simple example 2, based on the fact that when we remove a link it will not decrease the quantity of driver nodes. For example, when we remove the link from node *v*_4_ to node *v*_3_ in Fig. 2, according to the k-walk theory in Re. [8], it is easy to know that node *v*_1_ can control *v*_3_, *v*_4_ and *v*_6_. Therefore, the nodes {*v*_2_,*v*_4_} and {*v*_1_} as the optional driver nodes are ignored by the existed methods for the two networks

However, there are no existing methods to efficiently solve the problem. For example, in Fig. 1, for simple network 1 we want to control the target nodes *O* = {*v*_3_,*v*_4_, *v*_6_,*v*_7_} with the minimum the quantity of driver nodes and also expect to maximum the identified driver nodes within the constrained nodes *Q* = {*v*_2_,*v*_4_}. But Liu’s approach [6] and Gao’s approach [8] fail to find the optional driver nodes set for target controlling the *O* = {*v*_3_,*v*_4_,*v*_6_,*v*_7_} (see Fig. 1).To overcome the limitations of the existed approaches for the target control problem in complex networks, we develop a novel objective optimization algorithm (TCOA). The key consideration of our TCOA to solve the problem (4) is that 1) find the controllable targets of each network node without destroying target controllable of the whloe system; 2) to extract the optimal driver nodes of the network by using objectives-guided optimization with integer linear programming and Markov chain Monte Carlo (MCMC). The former is to guarantee the identified subset satisfying the constrained condition. And the latter is to guarantee the subset to be optimal for the two objectives.

### The framework of our TCOA

Our TCOA presents an algorithm for detecting driver nodes that can best control a network. We adopt the paradigm of local (linear) controllability. Different from the related works, our goal is not only to minimize the quantity of drivers, but also to maximize the quantity of drivers within a given pre-selected subset. The consideration for our TCOA is in the adding of different and more efficient strategies to find the optimal driver nodes of the graph. The TCOA algorithm consists of three steps: i) Identifying the controllable subsystem by constructing target control tree; ii) Finding the optional drivers with the Integer Linear Programming (ILP); iii) Further optimizing the driver set by using MCMC samplings. The details of TCOA are introduced in bellows.

#### Step 1 Identifying target controllable subspace by constructing target control tree

**Definition 1** *The target control tree is defined as a multi-layers network where the nodes in the bottom layer represent the target nodes and the nodes in the other layers consists of the upstream control nodes.*

Note that in our previous reseach [19] we have defined the upstream control nodes as the nodes which can control the target nodes in network *G*(*V*,*E*).To efficiently obtain the target control tree for controlling targets in a complex network, a algorithm (greedy algorithm) is designed to construct the target control tree as is listed in Table 1. Note that in our greedy algorithm, we use the “linking and dynamic graph” to represent the iterated bipartite graph. The relationship between maximum matching in the “linking and dynamic graph” and “the target control tree” can be explained as follow: at each iterated bipartite graph among the “linking and dynamic graph”, we can obtain the maximum matching, which result in a sub-graph; in the sub-graph, the maximum matching determines which paired nodes can be connected in “the target control tree”. The maximum matching of a given general bipartite graph can be efficiently obtained by using Hopcroft-Karp algorithm [20, 21]. Since Hopcroft-Karp algorithm runs in *O*($\sqrt{\mid \left|\mathrm{V}\right|\mid }$*||*E||*) time in the network *G*(*V*,*E*), the whole maximum complexity of the greedy algorithm is in *O*(r*$\sqrt{\mid \left|\mathrm{V}\right|\mid }$*||*E||*), where *r* is the iteration times when we obtain the updated bipartite graph.

**Table 1:** Our greedy algorithm for constructing target control tree for network *G*(*V, E*)

**Input:** Network *G(V, E),* target nodes *O*	
**Initialize:** *B*_0_ ←*(R*_0_, *L*_0_) //the right side *R*_0_ is made of target nodes *O*, and the left side *L*_0_ is made of nodes from which the targes could be reachable. *m*_*0*_←Matching(*B*_0_) //Find maximum matching *m*_*0*_ among bipartite graph *B*_0_ CF(*V*_0_, *m*_0_) ←Subgraph(*V*_0_, *m*_0_) // Let *V*_0_ = *R*_0_∪*L*_0_, we obtain a subgraph CF(*V*_0_, *m*_0_) **for** paired nodes (*v*_i_^0^*,v*_j_^0^)∈CF(*V*_0_, *m*_0_) **do //** if we could find a path from node *v*_i_^0^∈*L*_0_ to *v*_j_^0^∈*R*_0_, add edge *e*_k_^0^ *=* (*v*_i_^0^*,v*_j_^0^) to *TCT* . *E*_0_← *E*_0_∪*e*_k_^0^ **end** **While** *L*_*n*_≠ ∅ (*n* ≥ 1) **do** *R*_*n*_ ← *L*_*n-1*_ //Let the set of nodes in *L*_*n-1*_ to be the new *R*_*n*_ set *B*_*n*_ ←*(R*_n_, *L*_n_) //get a new bipartite graph *B*_*n*_. *m*_*n*_←Matching(*B*_n_) //Calculate a maximum matching *m*_*n*_ in *B*_*n*_ *V*_*n*_ *= V*_*n-1*_∪*L*_*n*_ CF(*V*_*n*_, *m*_*n*_) ←Subgraph(*V*_*n*_, *m*_*n*_) **for** paired nodes (*v*_i_^*n*^*,v*_j_^*n*^)∈CF(*V*_*n*_, *m*_*n*_) **do //** *v*_i_^*n*^∈*L*_n_, *v*_j_^*n*^∈*R*_*n*_ If there exists a directed path from *v*_i_^*n*^ to *v*_j_^*n*^_*,*_ add edge *e*_k_^*n*^ *=* (*v*_i_^*n*^*,v*_j_^*n*^) to *TCT*. Let *E*_n_ = *E*_n-1_∪(∪*e*_k_^n^). **End**	
**Output:** The target control tree, *TCT* ≡ (*V*_*TCT*_*, E*_*TCT*_) = (*V*_*n*_, *E*_*n*_)	

**Theorem (Target controllable subsystem identification theorem)** *In the target control tree, the target node v*_*j*_ *among the bottom layer could be controlled by the node v*_*i*_ *among the up layer if node v*_*j*_ *is accessible from node v*_*i*_ *.*

This result of our theorem (the details of proof are in *supplementary note* 1 of Additional file 1), can allow us to find the controllable targets of node *v*_*i*_, denoted by TCS(*v*_*i*_). The term “control” means that when we act control signals on the node *v*_*i*_, the nodes state in TCS(*v*_*i*_) can be changed to any stable or unstable targte state from any initial state at a finite time.Following on our theorem, in the target control tree, we can apply the Breadth First Search algorithm (BFS) [22, 23] to obtain the controllable targets of node *v*_*i*_,TCS(*v*_*i*_).The procedure of BFS is shown in Table 2, Obviously, the maximum complexity of the BFS algorithm for identifying the controllable targets of all nodes in the target control tree *TCT* ≡ (*V*_*TCT*_, *E*_*TCT*_), where *V*_TCT_ are the nodes and *E*_*TCT*_ are the edges in *TCT*, is in the order of *O*(||*V*_*TCT*_||*||*E*_*TCT*_*||*) [22, 23].

**Table 2:** Breadth First Search (BFS) algorithm for identifying target controllable subspace

**Input:** Target control tree *TCT* ≡ (*V*_*TCT*_*, E*_*TCT*_)*, node v*_*i*_	
**Initialize:** TCS(*v*_*i*_) = ∅ , *N*_0_ ={*v*_*i*_}; **While** *N*_*k*_≠ ∅ (*k* ≥ 1) Find the neighbor nodes of all nodes in *N*_*k-1*_ layer, denoted by *N*_*k*_; **end while** Add the reachable nodes in *R*_0_ to TCS(*v*_*i*_);	
**Output:** The target controllable subspace of node*v*_*i*_, TCS(*v*_*i*_)	

**Fig. 2:**
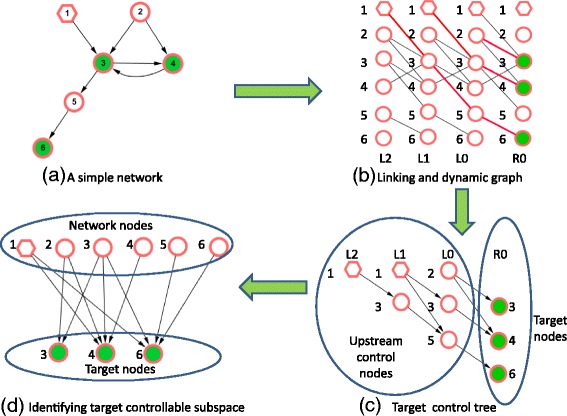
Demonstration of identifying the target controllable subspace **a** A directed network. The target control nodes set is {*v*_3_,*v*_4_,*v*_6_} (highlighted in green) and the constrained nodes set is {*v*_1_} (shape in hexagon). **b** Construct the “linking and dynamic graph”. Initialize a bipartite graph *B*_0_, where the right side *R*_0_ consists of the target nodes {*v*_3_,*v*_4_,*v*_6_}, and the left side *L*_0_ consists of the nodes that can reach the target nodes. Identify the maximum matching *m*_*0*_ *=* {(*v*_*2*_*,v*_*3*_)*,*(*v*_*3*_*,v*_*4*_)*,*(*v*_*5*_*,v*_*6*_)} in the initialized bipartite graph *B*_0_. Let the matched nodes {*v*_2_,*v*_3_,*v*_5_} in *L*_0_ to be *R*_1_ set and get a new bipartite graph *B*_1_. In the new bipartite graph *B*_1_, we can obtain the corresponding maximum matching *m*_*1*_ *=* {(*v*_*1*_*,v*_*3*_)*,*(*v*_*3*_*,v*_*5*_)}. Repeat this process and we obtain the maximum matching *m*_*2*_ *=* {(*v*_*1*_*,v*_*3*_)} in the new bipartite graph *B*_2_, which result in the “linking and dynamic graph” {*m*_*0*_*, m*_*1*_*, m*_*2*_} **c** Construct the target control tree from the “linking and dynamic graph”. In the sub-graph CF(*R*_0_ + *L*_0_,*m*0) and subgraph CF(*L*_0_ + *L*_1_,*m*_1_) and CF(*L*_1_ + *L*_2_,*m*_2_), add edges set *E*_*0*_ *=* {(*v*_*2*_*,v*_*3*_)*,* (*v*_*2*_*,v*_*4*_)*,* (*v*_*3*_*,v*_*4*_)*,*(*v*_*5*_*,v*_*6*_)} and *E*_*1*_ *=* {(*v*_*1*_*,v*_*3*_)*,* (*v*_*1*_*,v*_*5*_)*,* (*v*_*3*_*,v*_*5*_)} and *E*_*2*_ *=* {(*v*_*1*_*,v*_*3*_)} to *TCT*, which result in the target control tree. **d** We first form a new bipartite graph, in which the up layer consist of all the nodes in the network, and the bottom layer consist of the target nodes. Based on the target control subspace theorem, we can show that the node *v*_*i*_ in the *L*_*0*_*, L*_*1*_*, L*_*2*_ can control node *v*_*j*_ in *R*_*0*_, if there exist a path from *v*_*i*_ to the target node *v*_*j*_ in the target control tree *TCT*. And then we add edges from the node *v*_*i*_ in the up layer to node *v*_*j*_ in the bottom layer for the new formed bipartite graph. Finally we can identify the target controllable subspace of each node from the formed bipartite graph

**Fig. 3:**
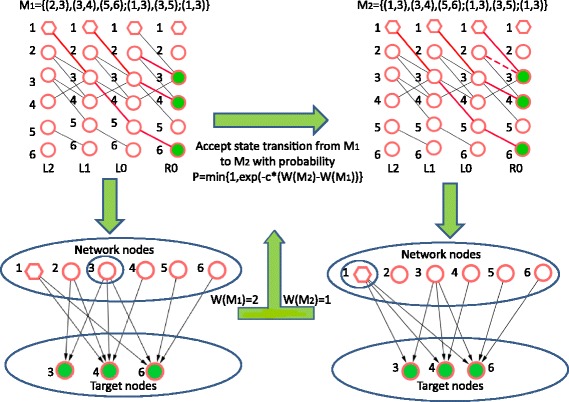
Demonstration of MCMC sampling. For the directed network in Fig. 2a, after two iterations, we first obtain the set of matched links (red edges) which form a Markov chain *M*_*1*_ in the “linking and dynamic graph”. Based on the Markov chain, we identify the target controllable sunspace of each node in the network; Then, we get the driver nodes {*v*_3_} by solving the problem (5) with integer linear programming (ILP), which result in the weight of the driver nodes W(M_1_) = 2.Then we generate a new Markov chain *M*_*2*_ by replacing the maximum matching in the *t* = 0 updated bipartite graph (*supplementary note* 2 of Additional file 1) and obtaining the new maximum matching in the later updated bipartite graph after *t* = 0. Based on the new Markov chain, we can identify the driver nodes {*v*_1_} with the weight W(M_2_) = 1. Finally according to the Metropolis-Hastings algorithm, we will accept the markov chain *M*_*2*_ with the probability *p*(*M*_*t*_*,M*_*t +* 1_) = min[1,exp.(c*W(*M*_*2*_)-c*W(*M*_*1*_)) for some c > 0, here we set *c* = 10

On the other hand, Liu et al. determined the controllable subsystem of any node in a network via linear programming [24], while Wang et al. propose a concept called control range to identify the controllable subsystem [25]. However, the existing two methods are still not efficient to identify the target controllable subsystem. In Figs. 2 and 3 we give an intuitive explanation to explain how we find the controllable targets of each network node. As shown in Fig. 2, we want to control the state transition of {*v*_3_, *v*_4_, *v*_6_}. By using Liu’s approach [24] and Wang’s approach [25], they both identify the controllable targets of *v*_1_ is {*v*_3_, *v*_4_ }. However, by using our method, the identified target controllable subsystem of node *v*_1_ is {*v*_3_, *v*_4_, *v*_6_} (see Figs. 2 and 3).

#### Step 2 Identifying the optional driver nodes by using integer linear programming

We first introduce an outlier measurement on a set of driver nodes that quantifies the quantity of driver nodes outside the pre-selected node-set,

$$ \mu (M)=\left\Vert D-Q\right\Vert =\sum \limits_{v_i\in U}{x}_i-\sum \limits_{v_j\in D}{y}_j $$

where *x*_*i*_ = 1 when a node *v*_*i*_ belongs to the driver set and ∑*x*_*i*_ denotes the quantity of driver nodes; *y*_*j*_ = 1 when node *v*_*j*_ belongs to the pre-selected set *D* and ∑*y*_*j*_ denotes the quantity of driver nodes in the pre-selected node-set. To take into account both the quantity of driver nodes and the quantity of driver nodes in the pre-selected set, we define the weight

$$ W(M)=\left\Vert D\right\Vert +\left\Vert D-Q\right\Vert =2\sum \limits_{v_i\in U}{x}_i-\sum \limits_{v_j\in D}{y}_j $$

Note that *W*(*M*) is only one candidate measurement of the trade-off between the quantity of driver nodes and the quantity of driver nodes in the pre-selected set. After obtaining the controllable targets of each network node and the weight of the driver node-set, the optional driver nodes guaranteeing the constrained target controllable can be approximately determined by the following Integer Programming model,

5a $$ \min W(M)=2\sum \limits_{v_i\in U}{x}_i-\sum \limits_{v_j\in D}{y}_j $$

5b $$ s.t.\sum \limits_{v_i\in {F}_u\ }{x}_i\ge 1\kern0.5em \left(\mathrm{every}u\in O\right) $$

5c $$ {x}_j={y}_j\left({v}_j\in D\subseteq U\right),{x}_j,{y}_j\in \left\{0,\kern0.5em 1\right\} $$

In the problem (5), the function (5a) is to get the optional driver node-set with the minimum quantity of driver nodes and the maximum quantity of driver nodes in the pre-selected nodes set. The constraint (5b) aims that at least one driver node could control a target node. The constraint (5c) points that the value of *y*_*j*_ is equal to that of *x*_*j*_ if node *v*_*j*_ belongs to the pre-selected set *Q*. In fact, the problem (5) could be efficiently solved for thousands of variables with a LP-based classic branch and bound method [26, 27].

#### Step 3 Optimizing the driver nodes by MCMC samplings

Here we define the maximum matching of the “linking and dynamic graph” as a Markov chain. The state space of the Markov chain is the set of all the possible maximum matching of the “linking and dynamic graph”. In our greedy algorithm, for a given node different sets of controllable targets could be found when we obtain different maximum matchings (e.g. the red edges in the Fig. 3). That is, for different Markov chains, the different sets of driver nodes with different weight *W*(*M*) could be obtained. The optimal different Markov chains need to be sampled from the state space, so that, a Markov Chain Mont Carlo method (MCMC) [28] is used. The MCMC approach samples sets of maximum matching, with the probability of sampling a set *M* proportional to the weight W(*M*) of the set. Thus, the frequencies of the maximum matching sets in the MCMC method provides a ranking of maximum matching sets, in which the sets are ordered by decreasing sampling frequency. The advantages will prove useful in analysis of real network data below.

The basic idea of MCMC, implying on our objective optimization, is to build a Markov chain whose states are the collections of *k* adjoin paths connecting to the target nodes in the “linking and dynamic graph” and to define transitions between states that differ by one target node. With the Metropolis-Hastings algorithm [29], we sample sets of maximum matching M⊆G of *k* adjoin paths in the iterated bipartite graph with a stationary distribution that is proportional to exp.(c*W(*M*)) for some *c* > 0, which gives a desired stationary distribution on the state space.The advantage will prove useful in analysis of real mutation data below. The MCMC method is described as follows:

**Initialization:** By using greedy algorithm, obtain the initial Markov chain *M*_*0*_;

**Iteration:** For *t* = 1, 2,…, obtain *M*_t + 1_ from *M*_*t*_ as follows,

- Choosing a path *w* uniformly at random in *M*_t_. Then choose randomly an edge *e*_k0_ inside the path. Delete the edge *e*_k0_ in the chosen bipartite graph *B*_k0_ among the “linking and dynamic graph”. For the augmenting path, when we alternate unmatched and matched links (*Supplementary note 2* in Additional file 1), we can obtain a new matched edge *e’*_k0_ in the chosen iteration *k*_*0*_, which result in a new matched path *v* among the “linking and dynamic graph”. Then a new Markov Chain M_t+1_ = M_t_ \ {w} ∪ {v} has been obtained.
- Accepting the new Markov chain *M*_t + 1_ with the probability *p*(*M*_*t*_,*M*_*t + 1*_) = min[1,exp.(c*W(*M*_*t + 1*_)-c*W(*M*_*t*_)), else reject it.

We will terminate the procedure of the MCMC sampling when the value of *W*(*M*) converge within 100 iteration time units. Otherwise, the search process is terminated if the iteration time exceeds the fixed default value *N*_max_ = 1000. We have explored different values of *c*, and also use *c* = 10 in numerical experiments, which we found empirically to give the best tradeoff between the exploration of different sets and the convergence to sets with high weight *W*(*M*) on the simulated data and the pancreatic cancer data. The effect of parameter on the convergence of weight *W*(*M*) on the simulated datasets and two biological networks are provided in *Supplementary note 3* and *Supplementary note 4* of Additional file 1*.*

### The complexity analysis of TCOA

The TCOA method contains three parts:

(i) For constructing the target control tree for the network *G*(*V,E*), we apply the developed greedy algorithm, to find the maximum matching of the “linking and dynamic graph”. In fact, the developed greedy algorithm runs in the order of $\mathrm{O}\left({\mathrm{r}}^{\ast }{\sqrt{\left\Vert \mathrm{V}\right\Vert}}^{\ast}\left\Vert \mathrm{E}\right\Vert \right)$, where *r* denotes the iteration times in the iterated bipartite graph.

(ii) In phase of finding the controllable targets of all network nodes, we apply the BFS algorithm to the constructed target control tree. Therefore the maximum complexity of the BFS algorithm for finding the controllable targets of all nodes is in the order of *O*(||*V*_*TCT*_||*||*E*_*TCT*_*||*), where *V*_*TCT*_ denotes the nodes and *E*_*TCT*_ represents the edges in target control tree *TCT* (*V*_*TCT*_ *= V,* ||*E*_*TCT*_*|| < =*||*E||*) .

(iii) For the phase of finding the optional driver nodes set, the optional driver nodes set is obtained by using the integer linear programming. Specifically, we used a branch-and-bound algorithm, an automatic method with a greedy *O*(log(||V||)) of solving discrete programming problems, as implemented by function *intlinprog* of the MATLAB programming language to solve our binary integer-programming problem [30]. To find more optional solutions, MCMC sampling method has been adopted, resulting in the overall complexity of our TCOA approach $\mathrm{O}\Big({\mathrm{m}}^{\ast}\left({\mathrm{r}}^{\ast }{\sqrt{\left\Vert \mathrm{V}\right\Vert}}^{\ast}\left\Vert \mathrm{E}\right\Vert +{\left\Vert \mathrm{V}\right\Vert}^{\ast}\left\Vert {\mathrm{E}}_{\mathrm{TCT}}\right\Vert +\log \left\Vert \mathrm{V}\right\Vert \right)$, which can be approximately considered as O(m^∗^‖V‖^∗^‖E_TCT_‖) where *m* is sampling number, and *E*_*TCT*_ is the edges set of the target control tree.

## Results

### Experiment results of real-world networks

To evaluate the target control efficiency on an arbitrary network, we first introduce two factors αand β, which represent the ratio of the target nodes and constrained (or pre-selected) nodes to the whole network nodes respectively; To target control the target nodes *O*(α), our TCOA can identify the optional nodes set *D*(*α*, *β*) with the minimum driver nodes and the maximum quantity of driver nodes contained in a given constrained set *Q*(β).‖*D*(*α*, *β*)‖/‖*O*(*α*)‖ denotes the ratio of the quantity of identified drivers to the quatity of targets, and ‖*D*(*α*, *β*) ∩ *Q*‖/‖*D*(*α*, *β*)‖ denotes the ratio of the quantity of identified drivers among constrained (or pre-selected) nodes to the quantity of all the identified drivers.

Then we introduce two target controllability parameters. One is the average ratio of drivers to targets,

$$ {E}_1={\int}_0^1{\int}_0^1\left(\left\Vert D\left(\alpha, \beta \right)\right\Vert /\left\Vert O\left(\alpha \right)\right\Vert \right) d\alpha d\beta $$

which reflects the cost of controlling targets in the complex network. And another parameter is the average ratio of constrained (or pre-selected) nodes in the drivers to all the drivers,

$$ {E}_2={\int}_0^1{\int}_0^1\left\Vert D\left(\alpha, \beta \right)\cap Q\right\Vert /\left\Vert D\left(\alpha, \beta \right)\right\Vert \mathrm{d}\;\alpha\;\mathrm{d}\;\beta $$

which reflects the verifiability of identified driver nodes in target controlling the network.

Note that whenα = 1, β = 1, both of *E*_1_ and *E*_2_ can be reduced to the fraction of drivers to control the full networks. When 0 < α < 1, β = 1, *E*_1_ is reduced to the fraction of driver nodes to control the target nodes, and *E*_2_ = 1. However in our paper, we focus on the problem that when 0 < α < 1, 0 < β < 1, how to identify optional driver nodes to minimize the measure *E*_1_ and to maximize the measure *E*_1_. In fact, we have selected α = 0.1,0.2,…,1 andβ = 0.1,0.2,…,1, and applied our TCOA to calculate the two target controllability parameters *E*_1_ and *E*_2_. We have obtained the data of the real-world networks from [7, 8], and for the convenience we provide the data description in the (Additional file 2: Table S1). The results on these real networks are listed in the Table 3.

**Table 3:** The properties of real networks

Network	*N*	*L*	*<k>*	*E* ^*1*^_1_	*E* ^*2*^_1_	*E* ^*3*^_1_	*E* ^*1*^_2_	*E* ^*2*^_2_	*E* ^*3*^_2_
*Regulatory*									
TRN-EC	418	519	1.24	0.8119	0.8230	0.7717	0.5577	0.5963	0.6082
Yeast	688	1079	1.57	0.8083	0.7314	0.7141	0.5460	0.5798	0.5880
*Foodweb*									
Chesapeake	39	177	4.54	0.3049	0.2331	0.2318	0.5521	0.7331	0.8374
ChesUpper	37	215	5.81	0.2777	0.2450	0.2164	0.5502	0.6999	0.8304
Florida	128	2106	16.45	0.2849	0.1923	0.1255	0.5383	0.7506	0.8390
*Electronic circuits*									
s420	252	399	1.58	0.3466	0.1761	0.1666	0.5484	0.7315	0.7434
s208	122	189	1.55	0.3499	0.2106	0.2021	0.5582	0.6967	0.7826
s838	512	819	1.60	0.3563	0.1821	0.1780	0.5438	0.6797	0.6899
*Airports*									
USAir97	332	2126	6.40	0.4410	0.3047	0.2870	0.5477	0.6545	0.6815
*Trust*									
colledge_student	32	96	3.00	0.3285	0.3382	0.2103	0.5387	0.7457	0.8649
*Words*									
glossGT	72	118	1.64	0.7010	0.6441	0.5630	0.5442	0.6192	0.6701
*Web*									
Polblogs	1490	19,090	12.81	0.5414	0.4605	0.4572	0.5953	0.6430	0.6459
*Genetic*									
Rattusp	2640	4268	1.62	0.7385	0.6808	0.6756	0.5990	0.6374	0.6397
Celegants	3879	8482	2.19	0.7250	0.6340	0.6315	0.5994	0.6370	0.6400
Plasmodium	1203	2522	2.10	0.5909	0.4489	0.4468	0.6044	0.6709	0.6766

Here, we list the network types, network name, quantity of nodes in network (*N*), quantity of edges in network (*L*), the average degree of network <*k*>, the average ratio of drives to the targets by using Gao’s method (*E*^*1*^_1_*)* and our TCOA without MCMC samplings (*E*^*2*^_1_) and our TCOA (*E*^*3*^_1_) respectively and average ratio of drivers within the set of constrained (or pre-selected) nodes to all the identified drivers by using Gao’s method (*E*^*1*^_2_*)* and our TCOA without MCMC samplings (*E*^*2*^_2_) and our TCOA (*E*^*3*^_2_) respectively. The more detail descriptions of the real-world networks including the network types, names and references, quantity of nodes and edges and brief description and the downloaded websites, are shown in Additional file 2: Table S1

Obviously, we can conclude that TCOA can efficiently identify the driver nodes with optional property to guarantee the system target controllable, compared to the existing method. However with increasing size of *Q*, our TCOA is receiving more and more guidance, and is expected to outperform Gao’s method, which does not take a constrained set as input. From the Table 3, we find our TCOA can not only find more driver nodes contained in the constrained nodes set *Q* but also detects the less quantity of driver nodes*.*

The novelty of our TCOA is the proposed analysis framework consisting of target control tree, ILP model and MCMC sampling for improving efficiency. In addition to the algorithm comparision between TCOA and other existing methods, we have also carried on more comparisons to investigate the contribution of ILP and MCMC in TCOA.To evaluate the advantage of the ILP and MCMC sampling, we list the result of our TCOA without MCMC sampling (only with ILP) and the result of our TCOA. From Table 3, we find that our TCOA can perform better than the TCOA only with ILP but without MCMC sampling, supporting strongly the efficiency of the MCMC sampling. We also find that our TCOA can achieve better results than Gao’s method even without MCMC sampling.

### Case studies on PPI signaling transduction networks in pancreatic cancer

As further evidences of the applicability of TCOA, we have carried TCOA on PPI signaling transduction networks in pancreatic cancer. The main cause of cancer is genetic and epigenetic alterations, which allow normal cells to over-proliferate as tumor cells [31]. To comprehensively understand the specificity in signaling networks, we have to understand how distinct pathways communicate with each other and how proteins of one pathway make interactions with related signaling components. Here, to understand the various information-processing abilities employed during the molecular alteration of the cancerous cells [32], we obtain directed PPI network of 1569 interactions from 991 nodes in pancreatic cancer. The directed PPI cancer data, uses SIGNOR (SIGnaling Network Open Resource) database [33], which outputs binary matrix representations for the used-provided protein lists and allows us to create directed graphs between signaling entities. The networks are available in Network Controlability Project [34] or seen (Additional file 3: Table S2). In our paper, in total 1507 approved proteins (or genes) by the Food and Drug Administration (FDA) have been selected as the constrained (or preselected) nodes in the directed PPI network which will have a prior-known molecule mechanism, see (Additional file 4: Table S3). As is well known, only a subset of these alterations called essential proteins, from the hundreds of genomic alterations in various biological pathways [35, 36], are driving the disease initiation and progression. In the Ref. [34], researchers collected essential gene data for all cancer from the COLT-Cancer database [37]. In particular, they considered the HPAF-II cell lines for pancreatic cancer, and follow the GARP (Gene Activity Rank Profile) and GARP-*P* value of corresponding proteins mentioned in the database. Since previous studies showed that proteins with lower GARP score are more essential and directly associated with oncogenesis [38], they selected only those essential proteins whose GARP value is in the negative range, and moreover, whose GARP-P value is less than 0.05 (p < = 0.05). Following the above criteria, they identified 168 essential proteins available in the SIGNOR PPI network database in pancreatic cancer are selected as the targets to be controlled by the input signals.The essential proteins data can be seen in (Additional file 5: Table S4).

Our TCOA focus on how to identify the optional driver proteins with the minimum quantity of drivers and the maximum of the constrained FDA-approved proteins, to control the essential target proteins. We have also applied Liu’s method [6] to control the full network and apply Gao’s method [8] and our method to obtain the driver nodes to target control the network. The results seen in Table 4, indicate that we can identify less quantity of driver nodes by using TCOA compared to Liu’s method [6] and Gao’s method [8]. Furthermore, among the driver nodes, we can also obtain more drug targetable nodes.

**Table 4:** The properties of detected driver nodes with Liu’s method, Gao’s method, and our method in Pancreatic cancer network

Method	Pancreatic cancer network		Inflammatory bowel disease network	
	f1	f2	f1	f2
Liu [6]	4.0952	0.0596	39.0208	0.0806
Gao [8]	0.8750	0.0748	0.7917	0.1316
Our method	0.4702	0.4302	0.1875	0.5506

The columns represent the following information per disease network: Different methods, the fraction of the quantity of drivers vs. the target nodes (f1), the fraction of the driver nodes within drug target nodes in FDA vs. the quantity of driver nodes (f2)

Furthermore, in *Supplementary note* 5 of Additional file 1*.*, we also give the capacity [39] and the corresponding clinical information of the identified driver proteins by using our algorithm TCOA. In the *Supplementary note* 5 of Additional file 1, we have analyzed the drug-targetable proteins identified by TCOA as part of the strategies to control the cancer essential proteins, and we found that most of the TOP-20 proteins could be a direct target in cancer therapy. We also look for anti-cancer drugs for the drug target proteins identified by TCOA, whose results are also listed in *Supplementary note 5* of Additional file 1. We find that in some cases they have been used in current cancer type-specific drugs and drug-therapies. Among the 42 identified driver genes, 34 of them have not been previously reported as the drug targets. This suggests that our TCOA will be very useful in identifying potential drug targets.

### Case studies on inflammatory bowel disease network from KEGG

The causes of the common forms of idiopathic Inflammatory bowel disease (IBD) remain unclear though considerable progress [40]. Here, we utilize the network, in KEGG [41] as is listed in (Additional file 6: Table S5). The network consits of 4798 nodes and 105,606 directed and undirected edges (or bi-directed edges). To identify the drug targets, in total 702 approved proteins (or genes) by the Food and Drug Administration (FDA) have been selected in the network from the Drug Bank database [42], see (Additional file 7: Table S6) as the constrained nodes set in our TCOA.

**Fig. 4:**
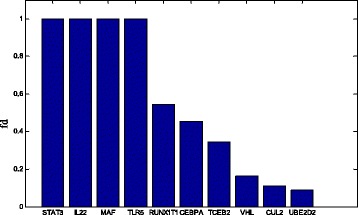
The frequency (fd) of the identified driver nodes within the constrained nodes set for MCMC samplings in IBD disease network

In this study, we consider the genes in the Inflammatory bowel disease (IBD) pathways which is listed in (Additional file 6: Table S5) as the target nodes in our TCOA. We apply both Gao’s algorithm [8] and our algorithm to analyze the target controllability of the network related with Inflammatory bowel disease (IBD). The results are shown in Table 4, and we find that the driver nodes for control the whole network is more than the target nodes and is not necessary to control the full network by using Liu’s algorithm [6]. Furthermore the quantity of driven nodes needed for the control of target genes is actually much smaller than that of Gao’s method [8] according to our method analysis. Our TCOA also found sets of driver nodes containing more drug targetable nodes, meanwhile Liu’s method [6] and Gao’s method [8] cannot detect drug targetable nodes as drivers, which indicate the applicability of TCOA. In addition, we calculate the frequency that each network node acts as a driver in the phase of MCMC sampling (or the control capacity [39]) as shown in Fig. 4. As is seen, STAT3, IL22, MAF and TLR5 has higher probability to be potential drivers to change the states of disease-related genes. Furthermore the existed researches have reported that IL-22 and TLR5 can be a therapy target for IBD [43]. These results suggest STAT3 and MAF can be future drug targets for IBD therapy.

### General topological properties of driver nodes in inflammatory pancreatic cancer network and bowel disease network

**Fig. 5:**
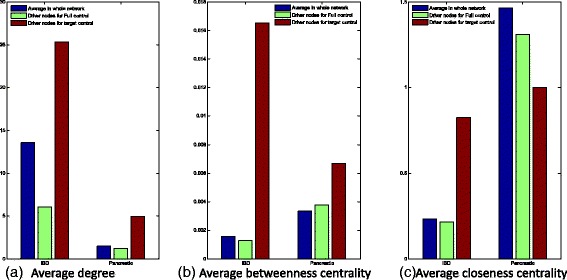
Topological properties of driven proteins in Pancreatic cancer network (a) Average out degree of driven proteins for our target control in compare to that in whole network (blue) and that of driver nodes for full control (green). (b) Average betweenness of driven proteins for our target control in compare to that in whole network (blue) and that of driver nodes for full control (green)

We have analyzed several topological properties of the drug-target proteins included by TCOA in the set of driven nodes. We calculated the average degree, the average betweenness centrality of these drug-target proteins by using our target control scheme, which are compared with Liu’s full control scheme [6] and the average values over the entire networks. We find that, in the disease networks, the drug-target driver nodes would have higher average degree than the average values over the entire networks as shown in Fig. 5a. This shows that the driver nodes tend to be high-degree nodes for target control the networks; In addition to the summarized results on the two biological networks, we have illustrated the network information of the validated results in (*supplementary note 6* of Additional file 1: Figure S5). From Additional file 1: Figure S5, we also found that the nodes with higher capacity have higher node degrees in the network and it also proved our statistic results in Fig. 5.

By using the full control scheme in the previous theoretical research [6], there exists an observation and conclusion that the driver nodes tend to be low-degree nodes as shown in Fig. 5a. However, for target control the biological networks, we interestingly find that the driver nodes tend to be high-degree nodes as shown in Fig. 5a, which is more consistent with the biological experimental results. The drug-target driven nodes also tend to have a higher average betweeness centrality as shown in Fig. 5b, which indicates that driver nodes would act as highly-traversed bridges in networks. By contrast, driver nodes display different weights on closeness centrality on particular disease network, as shown in Fig. 5c, which would mean the modularity around driver nodes would have significant rewiring in different conditions [32].

## Discussions

In fact, in our previous research, we studied another target control problem, called constrained target control (CTC) problem [19], which focuses on how to choose minimal drivers only within the set of constrained control nodes to change the states of the maximal targets. Different from CTC, we do not require that all the selected driver nodes must be in the constrained nodes set and our consideration for TCO has a double optimization to minimize the total quantity of driven nodes (on which a subsequent intervention is needed) and to maximize the percentage of constrained nodes among them (on which the findings are consistent with prior-known knowledge). Our results supply the novel insights into how we can efficiently target control a complex system, and especially many evidences on the practical strategic utility of TCOA to incorporate prior drug information into potential drug-target forecasts. However, this study is limited to focus on how to obtain the state transitions of the linear networks. It is more practical and necessary to target control the system with nonlinear dynamic in the future.

## Conclusions

It is rather difficult to study how to control a complex network, because we often do not know the true functional form of the underlying dynamics, such as biological networks. However, most systems operate near homeostasis, so in this study, we pose target control problem with objectives-guided optimization (TCO) and also provide a novel algorithm (TCOA) to study the local structural control of inherent nonlinear networks, which is more practical to target control the complex networks than the existing methods. In this work, the application of our new control tool TCOA provides more precision predictions compared to the existing methods on the study of structural target control of networks. Particularly, our work supports that the target control tools actually provide an efficient way to control a network through known drug-target nodes, in the cases of disease-associated networks. In addition, this work supplies a better understanding of the disease-associated biochemical networks and opens a new way to recover the drug-target based control mechanisms. This in turn could advance the future studies of various disease diagnostic techniques based on network, e.g., network biomarkers [44–47] and dynamic network biomarkers [48–52], efficient therapeutic approaches and personalized medicine [53].

## Additional files

Additional file 1: Supplementary material of A novel algorithm for finding optimal driver nodes to target control complex networks and its applications for drug targets identification. (DOC 2101 kb)

Additional file 2: Descriptions of real world networks. (XLSX 12 kb)

Additional file 3: FDA genes in directed PPI network. (XLSX 27 kb)

Additional file 4: Directed PPI interaction informations. (XLS 37 kb)

Additional file 5: Targets genes related with pancreatic. (XLSX 12 kb)

Additional file 6: KEGG 244-pathways network related with IBD disease. (XLSX 2411 kb)

Additional file 7: FDA genes in pathways network. (XLSX 18 kb)

## Abbreviations

CTC: Constrained target control
IBD: Inflammatory bowel disease
ILP: Integer linear programming
KEGG: Kyoto encyclopedia of genes and genomes
MCMC: Markov chain Monte Carlo
TCO: Target control problem with objectives-guided optimization
