# Supplementary material for: A novel algorithm for finding optimal driver nodes to target control complex networks and its applications for drug targets identification
Source: BMC Genomics. 2018 Jan 19;19(Suppl 1):924. doi: 10.1186/s12864-017-4332-z (PMC5780855; doi:10.1186/s12864-017-4332-z)
Supplement: Supplementary file 1 — Supplementary material of A novel algorithm for finding optimal driver nodes to target control complex networks and its applications for drug targets identification. (DOC 2101 kb) [file 12864_2017_4332_MOESM1_ESM.doc]

# Supplementary material of

# A novel algorithm for finding optimal driver nodes to target control complex networks and its applications for drug targets identification

Wei-Feng Guo^1^, Shao-Wu Zhang^1^*, Qian-qian Shi^2^, Cheng-Ming Zhang^2^ ,Tao Zeng^2^*, Luonan Chen^1,2,3^*

*^1^ Key Laboratory of Information Fusion Technology of Ministry of Education, School of Automation, Northwestern Polytechnical University, Xi’an 710072, China*

*^2^ Key Laboratory of Systems Biology, Innovation Center for Cell Signaling Network, Institute of Biochemistry and Cell Biology, Shanghai Institutes for Biological Science, Chinese Academy Science, Shanghai 200031, China*

*^3^Collaborative Research Center for Innovative Mathematical Modelling, Institute of Industrial Science, University of Tokyo, Tokyo 153-8505, Japan*

*** Corresponding author. Email: [zhangsw@nwpu.edu.cn](mailto:zhangsw@nwpu.edu.cn), [zengtao@sibs.ac.cn](mailto:zengtao@sibs.ac.cn),[lnchen@sibs.ac.cn](mailto:lnchen@sibs.ac.cn)

## **Supplementary Note 1 .** **The Proof of** T**arg**et controllable subsystem identification theorem

**Definition S1 (inaccessibility)**[^1^](#_ENREF_8) A state vertex *v_i_* in the digraph *G*(*V,E*) is called accessible iff there exists a directed paths reaching *v_i_* from the input vertices (origins), otherwise it is in accessible.

**Definition S2 (dilation)** [^1^](#_ENREF_8) The digraph *G*(*V,E*) contains no dilation iff there exists an edge subset $E^{*}\subseteq E$ so that no two edges in the subgraph of *G*(*V,E*), $CF(E^{*})=(V,E^{*})$, share a common starting vertex.

**Lemma (Lin’s Structural Controllability Theorem)** [^1^](#_ENREF_8) A linear control system *G*(*V,E*) is structurally controllable, if the digraph *G*(*V,E*) contains no inaccessible nodes and no dilation.

**Definition S3** **(target control configuration)** The target control configuration is also a subgraph of *G*(*V,E*) and is defined as $CF(V,M)=(V,M)$,where$M$denotes the Markov chain which consist of the set of matched edges in the updated bipartite graph.

**Theorem (Target controllable subsystem identification theorem)** The node *v_i_* in the **up layer** of the target control tree can control the target node *v_j_* in the **bottom layer** if there exist a directed path from the node *v_i_* to node *v_j_*.

Proof:

Obviously, there exists a directed path from node *v_i_* in the **up layer** of the target control tree to *v_j_* in the target control configuration$CF(V,M)$.Furthermore in the control configuration$CF(V,M)$, we have delete the possible existed dilations in each updated bipartite graph. Therefore there is no dilation to control node *v_i_* by applying control signals to node in the **up layer** of the target control tree. According to lemma[^1^](#_ENREF_8), nodes in the **up layer** of the target control tree can control *v_i_* in target control configuration$CF(V,M)$. According to Liu’s theorem[^2^](#_ENREF_9), adding edges will not affect the structural controllability of the system. Therefore, we can conclude that nodes in the **up layer** of the target control tree can control node *v_i_* in the network$G(V,E)$.

## **Supplementary Note 2 :Finding different maximum matchings in a bipartite graph**

Matched edges in the linking and dynamic graph constructed by our Greedy algorithm, are identified by the maximum matching in each updated bipartite graph. Therefore to generate different matched edges, we turn to the question how we find different maximum matchings in a bipartite graph. To generate a new maximum matching in a bipartite graph, we usually attempt to increase the matching size via an augmenting path that begins at a matched node, ends at an unmatched node and alternates between unmatched and matched links on the path. The procedure is in detail described as follows,

1. Initialize the starting maximum matching$m_{1}$;
2. Then randomly delete one matched link and identify the unmatched nodes on the

left and right size respectively; After removing one matched links ,we can identify the augmenting path that begins at the unmatched node on the left and ends at the unmatched node on the right;

1. Finally, by alternating between unmatched and matched links on the augmenting path, we can obtain a new maximum matching$m_{1}^{'}$.

For example, for the network in Figure S1 (a), a bipartite graph that is separated into the *out* sets (left side) and *in* sets (right side) is constructed in Figure S1 (b).The red links are matched, the black links are unmatched, and the matched link set forms a maximum matching *m_1_*. Proceeding from this maximum matching, we randomly choose node *v_2_* and leave the current matched vertices and links unchanged. Similar to Wang’s method[^35^](#_ENREF_35), we delete only the matched link *l_1,2_*. Then, we can identify an augmenting path that begins at the unmatched node on the left *v_1_* and ends at the unmatched node on the right *v_3_*; finally by alternating between unmatched and matched links on this augmenting path, we obtain a new maximum matching *M*_2_, as shown in Figure S1 (c). By contrast, because it needs to find augmenting path between the relevant matched node *v_1_* and ends at the presently unmatched node *v_2_*, Jia’s method[^3^](#_ENREF_14) and Wang’s method [^4^](#_ENREF_35) cannot produce any new maximum matching from the given *m*_1_. The difference is that we also enumerate all alternative maximum matchings that include all other elements except finding augmenting path that begins at the unmatched node on the left and ends at the unmatched node on the right.


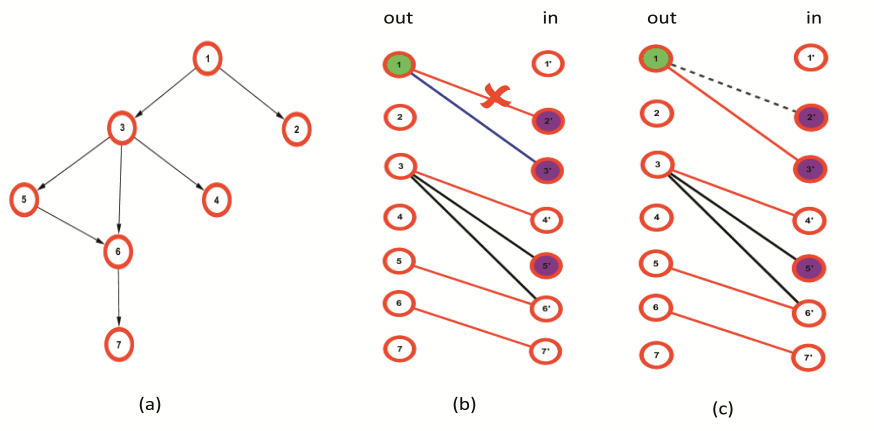


Figure S1 (a).A directed network. (b): A bipartite graph separated into the out and in sets; the red link set forms a maximum matching, *M_1_*, and the blue path is an augmenting path when the matched link *l_1,2_* is removed. (c): A new maximum matching *M_2_* constructed by alternating the blue augmenting path.

## **Supplementary Note 3 :The effect of different parameters on the result for 15 different real networks**

Figure S2 The effect *of* different parameters *c* on the defined weight of the identified driver nodes in nineteen real networks. The information of the fifteen real networks is provided in Table 3 and Table S1.

## **Supplementary Note 4: The effect of different parameters on the result for IBD network**

Figure S3 The effect *of* different parameters *c* on the defined weight of the identified driver nodes in pancreatic cancer networks.

Figure S4 The effect *of* different parameters *c* on the defined weight of the identified driver nodes in Inflammatory bowel disease network

## **Supplementary Note 5 :The clinical information of identified driver nodes on PPI signaling transduction networks in Pancreatic cancer**

**Table S1** The frequency of the identified driver nodes in pancreatic cancer network

| Rank | Driver | capacity | Anti-cancer drug | Rank | Driver | capacity | Anti-cancer drug |
| --- | --- | --- | --- | --- | --- | --- | --- |
| 1 | ERBB2 | 1 | Lapatinib | 22 | CDK6 | 1 | None |
| 2 | SRC | 1 | Dasatinib, Bosutinib,Ponatinib | 23 | DLG4 | 1 | None |
| 3 | PRKDC | 1 | None | 24 | FOS | 1 | None |
| 4 | MTOR | 1 | Temsirolimus | 25 | FYN | 1 | None |
| 5 | JAK2 | 1 | Ruxolitinib, Erlotinib | 26 | HDAC1 | 1 | None |
| 6 | CDK2 | 1 | None | 27 | HRH1 | 1 | None |
| 7 | AKT1 | 1 | None | 28 | LYN | 1 | None |
| 8 | GSK3B | 1 | Alectinib | 29 | NRP1 | 1 | None |
| 9 | ABL1 | 1 | None | 30 | PIM1 | 1 | None |
| 10 | IGF1R | 1 | None | 31 | TLR4 | 0.7612 | None |
| 11 | HDAC3 | 1 | Vorinostat | 32 | TOP1 | 0.6451 | None |
| 12 | RAF1 | 1 | None | 33 | EGFR | 0.5483 | None |
| 13 | INSR | 1 | None | 34 | CCND1 | 0.4838 | None |
| 14 | RAC1 | 1 | None | 35 | EGF | 0.4000 | None |
| 15 | PDPK1 | 1 | None | 36 | ERBB4 | 0.2580 | None |
| 16 | RET | 1 | Cabozantinib | 37 | HDAC2 | 0.2580 | None |
| 17 | GRB2 | 1 | None | 38 | MET | 0.1935 | None |
| 18 | ADCY1 | 1 | None | 39 | NTRK2 | 0.0645 | None |
| 19 | ALK | 1 | None | 40 | TGFB1 | 0.0645 | None |
| 20 | BTK | 1 | None | 41 | NTRK1 | 0.0258 | Sorafenib,Sunitinib,Pazopanib |
| 21 | CDK4 | 1 | Lapatinib | 42 | IL1R1 | 0.0064 | None |

The columns represent the following information per cancer network: the rank of the driver proteins based on the value of their capacity, the name of the driver proteins, the value of capacity, name of anti-cancer drug.

## **Supplementary Note 6 : The illustrative network examples in the two biological networks**

Figure S5 The illustrative network examples in the two biological networks

# **References**

1 Lin, C. T. Structural controllability. *Automatic Control, IEEE Transactions on* **19**, 201-208 (1974).

2 Liu, Y.-Y., Slotine, J.-J. & Barabási, A.-L. Controllability of complex networks. *Nature* **473**, 167-173 (2011).

3 T. Jia, A.-L. Barabási, Control capacity and a random sampling method in exploring controllability of complex networks, Scientific reports, 3 (2013).

4 B. Wang, L. Gao, Y. Gao, Y. Deng, Y. Wang, Controllability and observability analysis for vertex domination centrality in directed networks, Scientific reports, 4 (2014).
